# Supplementary material for: Global mapping of GalNAc-T isoform-specificities and O-glycosylation site-occupancy in a tissue-forming human cell line
Source: Nat Commun. 2022 Oct 21;13:6257. doi: 10.1038/s41467-022-33806-8 (PMC9587226; doi:10.1038/s41467-022-33806-8)
Supplement: Supplementary file 1 — Supplementary Information [file 41467_2022_33806_MOESM1_ESM.pdf]

## Supplementary Information for:

Global mapping of GalNAc-T isoform-specificities and O-glycosylation site-occupancy in a tissue-forming human cell line

Mathias I. Nielsen**, Noortje de Haan**, Weston Kightlinger, Zilu Ye, Sally Dabelsteen, Minyan Li, Michael C. Jewett , Ieva Bagdonaite, Sergey Y. Vakhrushev*, and Hans H. Wandall*

*To whom correspondence should be addressed. E-mail: hhw@sund.ku.dk and seva@sund.ku.dk

** Equally contributing authors.

### This PDF file includes:

Supplementary Figures 1 to 4

# Supplementary Figure 1

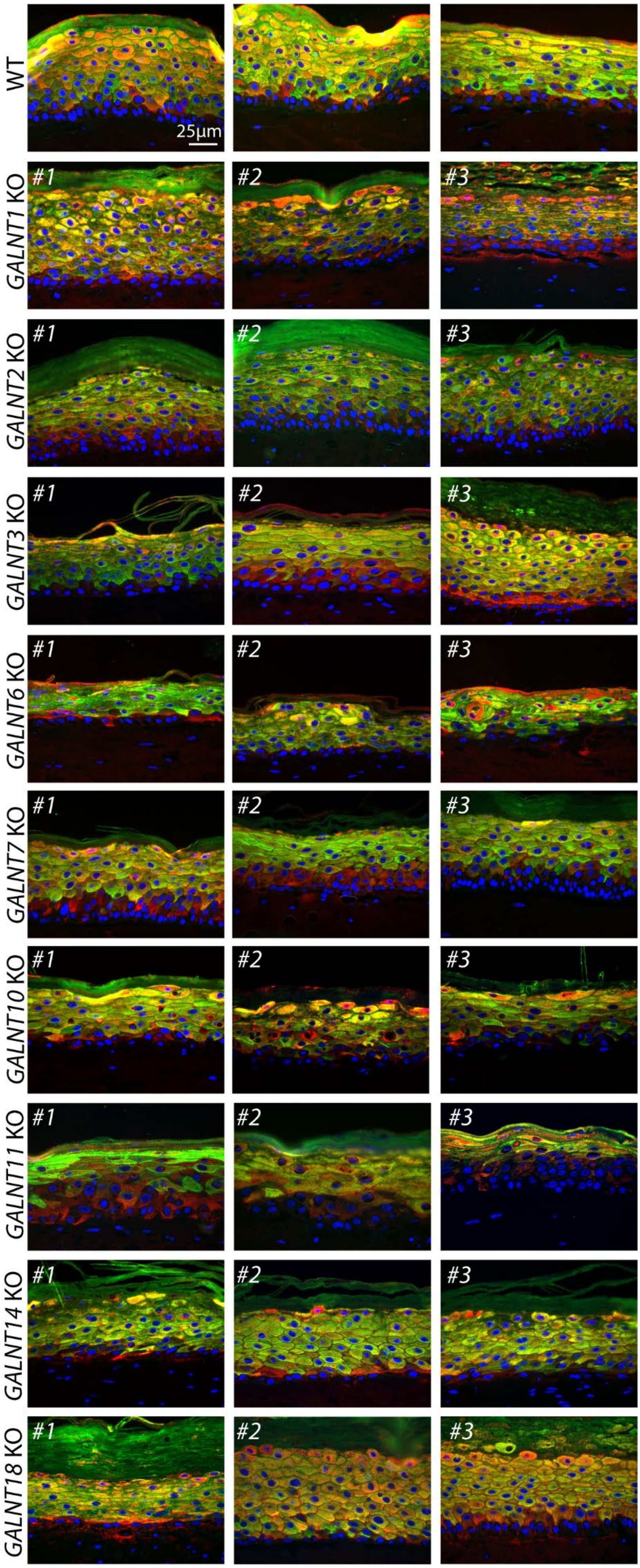


**Supplementary Figure 1:** Micrographs show sections of organotypic cultures (OCT) made with three different clones of *GALNT* KO tissues. At least three independent experiments were performed for each clone at different time points resulting in organotypic cultures with similar morphologies. Sections are stained with differentiation markers Keratin10 (green) and Involucrin (red). Nuclei labelled with DAPI (blue). Scalebar = 25µm.

# Supplementary Figure 2

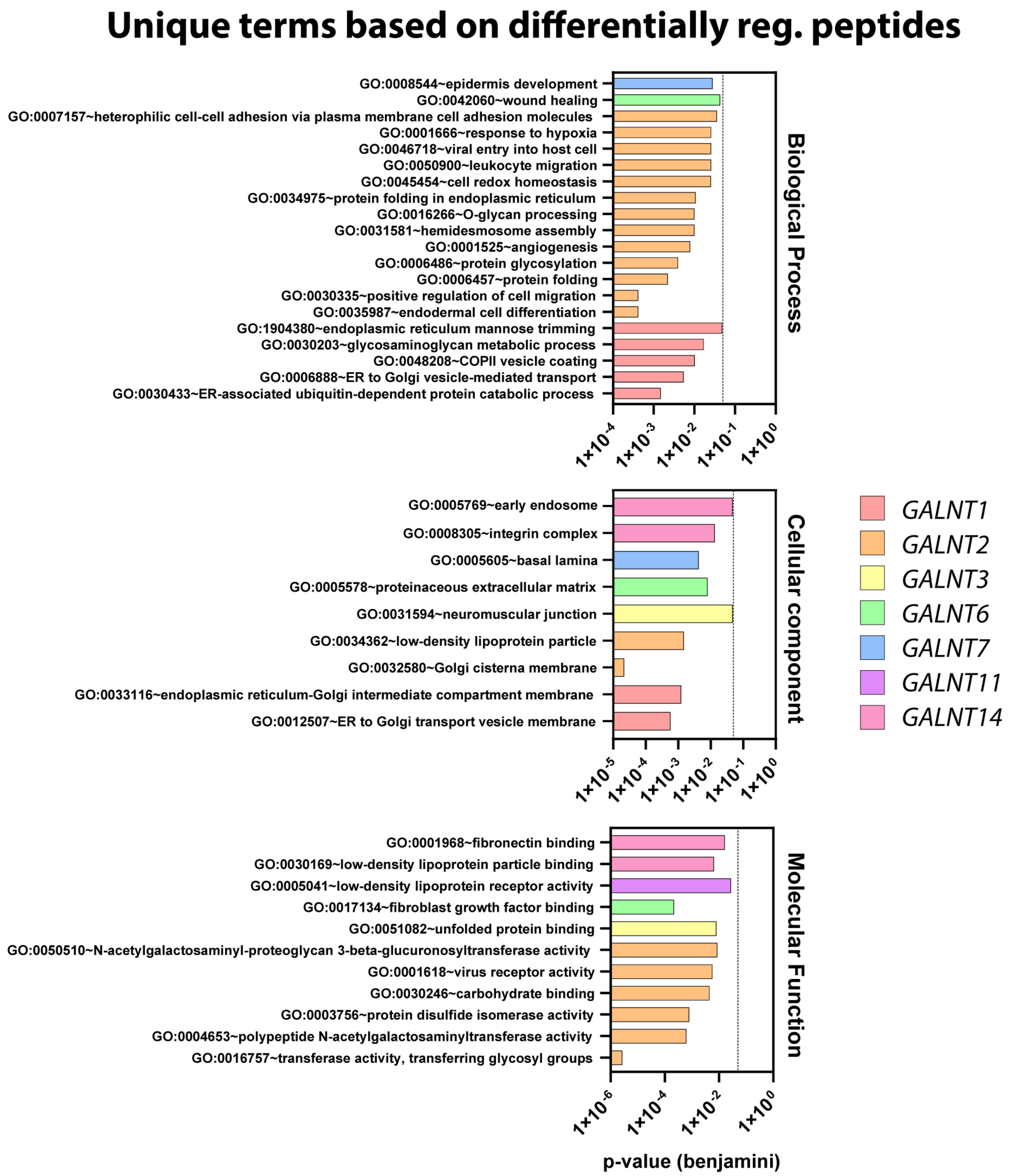


**Supplementary Figure 2:** Gene Ontology (GO)-term analysis of differentially glycosylated protein identifications (DAVID, q≤0.05, Benjamini adjusted). Each color represents differential GO terms unique to one of the *GALNT* differential datasets. Pvalue (Benjamini multiple comparison adjusted) of 0.05 is marked with a dashed line. Additional information can be found in Supplementary Figure 2 associated table below.

## Supplementary Figure 2 associated table. Additional GO-term analysis information

| GALNT | Category | Term | Count | Pvalue | Genes | Benjamini |
|---|---|---|---|---|---|---|
| **T1** | BP | GO:0030433~ER-associated ubiquitin-dependent protein catabolic process | 6 | 5.34E-06 | Q9UBV2, P11021, Q13438, P14625, Q9UKM7, O94905 | 0.001487 |
| **T1** | BP | GO:0006888~ER to Golgi vesicle-mediated transport | 7 | 5.85E-05 | Q8NI22, P49257, Q5JRA6, Q15363, P49755, P15514, Q9Y4L1 | 0.005432 |
| **T1** | BP | GO:0048208~COPII vesicle coating | 5 | 0.000128 | Q8NI22, P49257, Q15363, P49755, P15514 | 0.010158 |
| **T1** | BP | GO:0030203~glycosaminoglycan metabolic process | 4 | 0.000245 | P35052, P18827, Q4G148, P98160 | 0.01703 |
| **T1** | BP | GO:1904380~endoplasmic reticulum mannose trimming | 3 | 0.000776 | Q9UBV2, Q13438, Q9UKM7 | 0.048026 |
| **T1** | CC | GO:0012507~ER to Golgi transport vesicle membrane | 5 | 5.85E-05 | Q8NI22, P49257, Q15363, P49755, P15514 | 0.000581 |
| **T1** | CC | GO:0033116~endoplasmic reticulum-Golgi intermediate compartment membrane | 5 | 0.000132 | Q8NI22, P49257, Q15363, P49755, P15514 | 0.001233 |
| **T2** | BP | GO:0035987~endodermal cell differentiation | 6 | 2.03E-06 | P08648, Q13751, Q16787, P50281, P08572, P02751 | 0.000419 |
| **T2** | BP | GO:0030335~positive regulation of cell migration | 11 | 2.06E-06 | P08648, Q13753, P00749, P11021, P50281, O00592, P13726, P43121, P21709, P07996, O00622 | 0.000419 |
| **T2** | BP | GO:0006457~protein folding | 10 | 1.33E-05 | P11142, Q8NBS9, Q9BS26, Q96KC8, P14625, P13667, P27824, Q14696, P14314, P30101 | 0.002199 |
| **T2** | BP | GO:0006486~protein glycosylation | 8 | 3.21E-05 | Q96FL9, Q9HCN8, O60513, Q93063, O60512, Q9H6U8, Q8NCL4, Q10471 | 0.003976 |
| **T2** | BP | GO:0001525~angiogenesis | 10 | 7.16E-05 | P08648, P29279, P50281, P43121, O60462, P08572, P21709, P35354, P78504, P02751 | 0.007881 |
| **T2** | BP | GO:0031581~hemidesmosome assembly | 4 | 0.000101 | Q13753, Q13751, Q16787, Q9UMD9 | 0.010029 |
| **T2** | BP | GO:0016266~O-glycan processing | 6 | 0.000111 | Q96FL9, Q14435, Q8NCL4, Q7Z7M9, Q8NBI6, Q10471 | 0.010029 |
| **T2** | BP | GO:0034975~protein folding in endoplasmic reticulum | 4 | 0.000131 | P11021, P14625, P27824, P30101 | 0.010826 |
| **T2** | BP | GO:0045454~cell redox homeostasis | 6 | 0.000362 | Q8NBS9, Q9BS26, P13667, Q9BQE4, P30101, P07237 | 0.025605 |
| **T2** | BP | GO:0050900~leukocyte migration | 7 | 0.000421 | P08648, Q9Y624, O00592, P08195, P16070, P02751, P05026 | 0.025726 |
| **T2** | BP | GO:0046718~viral entry into host cell | 6 | 0.000432 | P08648, Q9Y624, P01130, Q92692, Q14118, P18564 | 0.025726 |
| **T2** | BP | GO:0001666~response to hypoxia | 8 | 0.000441 | P00749, P50281, P19021, P14625, Q92544, Q14571, P07996, P05026 | 0.025726 |
| **T2** | BP | GO:0007157~heterophilic cell-cell adhesion via plasma membrane cell adhesion molecules | 5 | 0.000653 | Q9NT68, P08648, Q9P273, Q92692, Q9NQS3 | 0.035966 |
| **T2** | CC | GO:0032580~Golgi cisterna membrane | 8 | 2.1E-06 | O60513, Q86X52, Q14435, O60512, Q8IZ52, O00461, Q9P2E5, Q10471 | 2.18E-05 |
| **T2** | CC | GO:0034362~low-density lipoprotein particle | 4 | 0.000158 | P01130, Q86X29, Q92673, Q9BQE4 | 0.001485 |
| **T2** | MF | GO:0016757~transferase activity, transferring glycosyl groups | 9 | 2.21E-08 | Q96FL9, O60513, Q93063, O60512, Q9H6U8, Q8NCL4, Q8N3Y3, Q8NBI6, Q10471 | 2.7E-06 |
| **T2** | MF | GO:0004653~polypeptide N-acetylgalactosaminyltransferase activity | 5 | 1.52E-05 | Q96FL9, Q14435, Q8NCL4, Q7Z7M9, Q10471 | 0.00062 |
| **T2** | MF | GO:0003756~protein disulfide isomerase activity | 5 | 2.27E-05 | Q8NBS9, Q9BS26, P13667, P30101, P07237 | 0.000792 |
| **T2** | MF | GO:0030246~carbohydrate binding | 9 | 0.000146 | Q96FL9, Q14435, Q13438, Q8NCL4, Q7Z7M9, P27824, Q12907, Q9UBG0, Q10471 | 0.004467 |
| **T2** | MF | GO:0001618~virus receptor activity | 6 | 0.000211 | P08648, Q9Y624, P01130, Q92692, Q14118, P18564 | 0.005711 |
| **T2** | MF | GO:0050510~N-acetylgalactosaminyl-proteoglycan 3-beta-glucuronosyltransferase activity | 3 | 0.000355 | Q86X52, Q8IZ52, Q9P2E5 | 0.008661 |
| **T3** | CC | GO:0031594~neuromuscular junction | 3 | 0.005864 | P05556, P05067, O75096 | 0.04728 |
| **T3** | MF | GO:0051082~unfolded protein binding | 5 | 0.000106 | P30533, P11021, P14625, P27824, P50454 | 0.007944 |
| **T6** | BP | GO:0042060~wound healing | 5 | 0.000384 | P18827, Q5JRA6, Q9NYQ6, P22223, P31431 | 0.043189 |
| **T6** | CC | GO:0005578~proteinaceous extracellular matrix | 7 | 0.000824 | P35052, P23471, P19957, Q9UMD9, Q92896, Q16610, Q9NZU0 | 0.007984 |
| **T6** | MF | GO:0017134~fibroblast growth factor binding | 5 | 2.13E-06 | P35052, Q14512, P06756, P22607, Q92896 | 0.000217 |
| **T7** | BP | GO:0008544~epidermis development | 4 | 0.000335 | P22528, P12272, Q9UMD9, Q9UBC9 | 0.028037 |
| **T7** | CC | GO:0005605~basal lamina | 3 | 0.000253 | O00468, P02751, P14543 | 0.004258 |
| **T11** | MF | GO:0005041~low-density lipoprotein receptor activity | 2 | 0.002309 | P01130, Q07954 | 0.027704 |
| **T14** | CC | GO:0008305~integrin complex | 3 | 0.001085 | P05556, P23229, P26006 | 0.013345 |
| **T14** | CC | GO:0005769~early endosome | 4 | 0.008093 | Q5VW38, P01130, O14786, Q92673 | 0.047401 |
| **T14** | MF | GO:0030169~low-density lipoprotein particle binding | 3 | 0.000383 | P01130, Q92673, P07996 | 0.006434 |
| **T14** | MF | GO:0001968~fibronectin binding | 3 | 0.00117 | P05556, P07996, P26006 | 0.016374 |

# Supplementary Figure 3

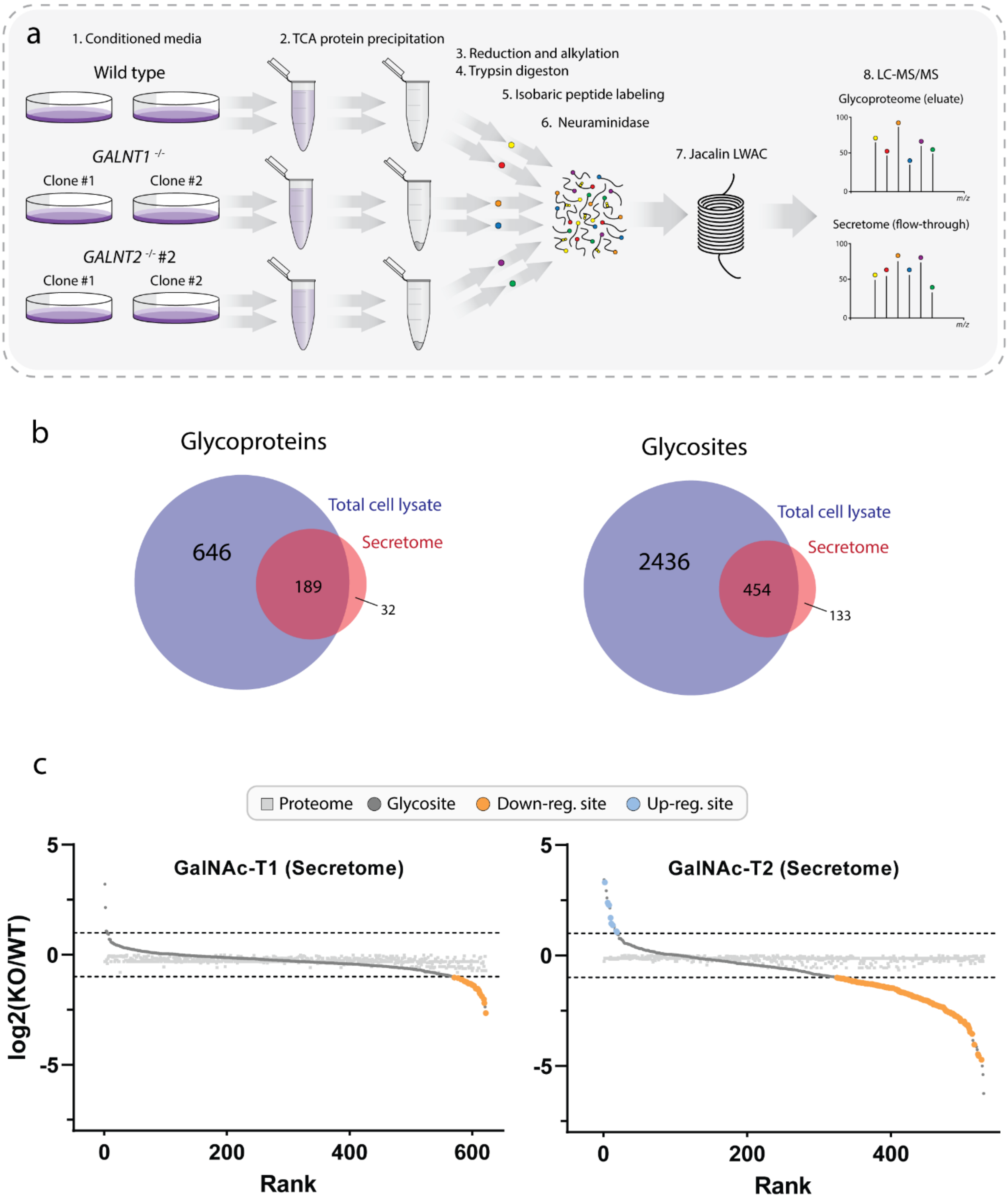


**Supplementary Figure 3:** Differential N/TERT-1 secretomes. **a)** Workflow summary for preparation on differential glycosecretomes. Conditioned media was harvested from N/TERT-1 WT and *GALNT1* and *-T2* knockout cells, and secreted proteins were isolated using trichloroacetic acid (TCA) precipitation. Secreted protein samples were then subjected to a similar glycoproteomic workflow as described in Figure 1A. **b)** Comparison of glycoproteins and glycosites identified in total cell lysate samples (blue) and secretome samples (red). **c)** Rank curve plot showing change in secretome glycopeptide (dark grey circles) abundances for *GALNT1* and *-T2* knockouts. Orange circles represent significantly down-regulated glycopeptides ($p$-value < 0.05 and log2 fold change < -1) and blue circles represent significantly up-regulated glycopeptides ($p$-value < 0.05 and log2 fold change >1). Sites from proteins with overall abundance change in the proteome (light grey squares) were removed. Source data are in Supplementary Data 2.

# Supplementary Figure 4

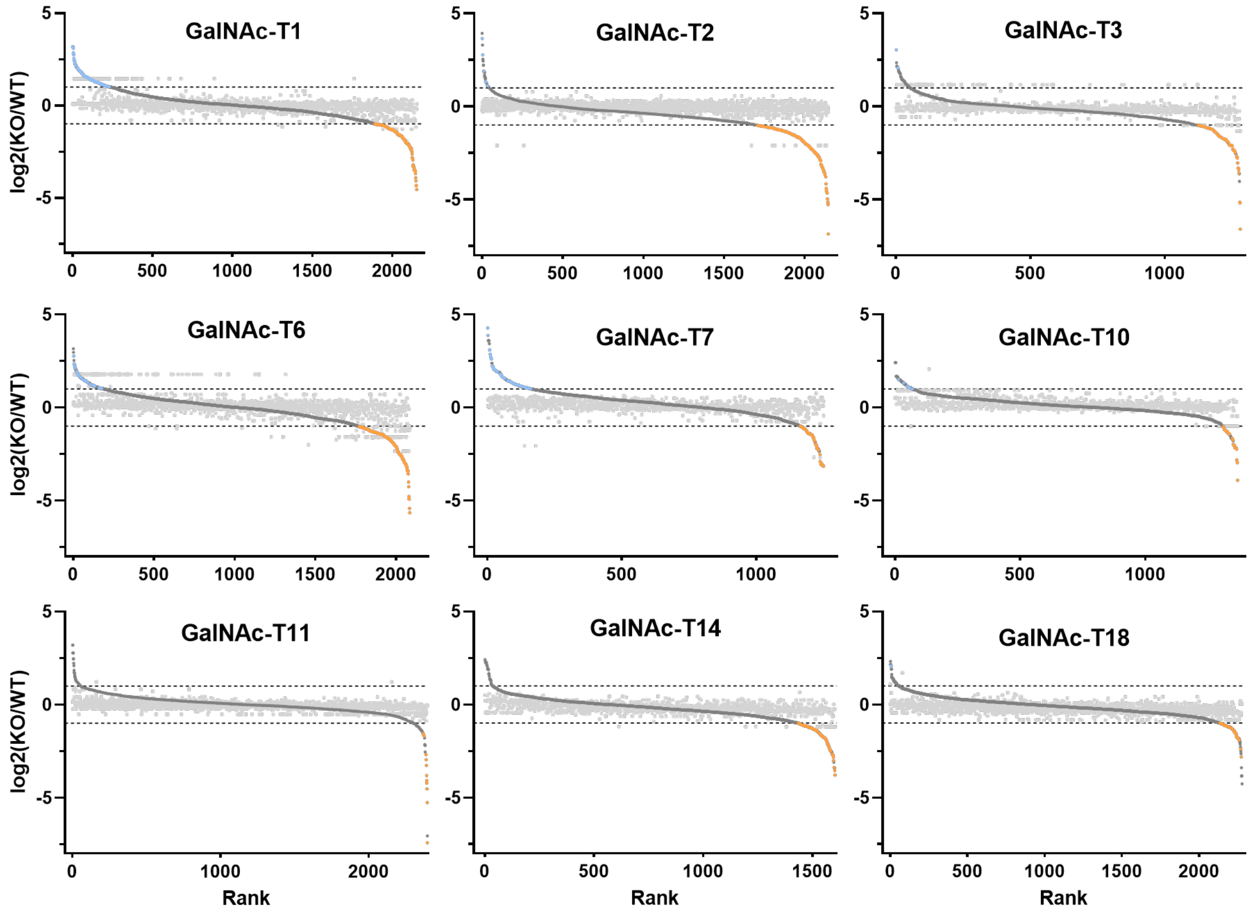


**Supplementary Figure 4:** Rank curve plot showing change in glycopeptide (dark grey circles) abundance for each of the 9 *GALNT* knockouts. Orange circles represent significantly down-regulated glycopeptides (*p*-value < 0.05 and log2 fold change < -1) and blue circles represent significantly up-regulated glycopeptides (*p*-value < 0.05 and log2 fold change >1). For each of the nine targeted *GALNT* genes, knockouts (n = 3 biologically independent samples) were compared in a multiplexed experiment with wild type material (n = 3 technically independent samples). A two-sided Student's t-test was used as a measure of statistical confidence and the *p*-values were adjusted for multiple testing, using an FDR of 5%. Source data are in Supplementary Data 2 and 4.
