# Supplementary material for: Global mapping of GalNAc-T isoform-specificities and O-glycosylation site-occupancy in a tissue-forming human cell line
Source: Nat Commun. 2022 Oct 21;13:6257. doi: 10.1038/s41467-022-33806-8 (PMC9587226; doi:10.1038/s41467-022-33806-8)
Supplement: Supplementary file 3 — Description of Additional Supplementary files [file 41467_2022_33806_MOESM3_ESM.docx]

**File Name:** Supplementary Data 1

**Description:** Sequence information of GALNT KO clones. All nine GalNAc-Ts expressed in N/TERT-1 WT cells were individually targeted for genetic knockout. Three distinct clones from each knockout were expanded. The desired genetic change was confirmed using indel-detection by amplicon analysis (IDAA) and DNA sequencing.

**File Name:** Supplementary Data 2

**Description:** Quantitative O-GalNAc glycoproteomics comparing the N/TERT-1 GALNT KOs vs. WT cells. TMT-based quantitative O-GalNAc glycoproteomics was performed for each of the GALNT KOs on three technical replicates of the N/TERT-1 WT cells (TMT126, 127 and 128), as well as three different clones of the respective GALNT KO (TMT129, 130 and 131), after sialidase treatment and jacalin LWAC enrichment. All glycopeptides listed per experiment had a ptmRS-determined probability score > 95 indicating the certainty of glycan localization within the peptide based on ETD. Abundance ratios obtained by HCD compared GALNT KO/WT. Glycopeptides with an abundance ratio <0.5 / >2 and an adjusted p-value < 0.05 were considered to be significantly different, but only if no change was observed in the abundance of the carrier protein (see Supplementary Data 4). Each entry represents a unique glycan site in the glycoproteome. These sites can be present simultaneously on the same peptide portion, resulting in replicated abundance values throughout the tables. “Glycosite” indicates the gene name of the protein and the amino acid (number) carrying the glycan. “GlyNum” indicates the total number of glycan sites on the identified glycopeptide. “GlycanType” indicates the identity of the glycan on the indicated site, while “Modi” summarizes the complete set of glycans present on the glycopeptide. The “target list” per GALNT KO lists the single-site glycopeptides that were significantly downregulated in the respective KO cells. A regulated site was indicated as “specific” when only downregulated by the KO of one of the GalNAc-Ts. MS data files are available via the ProteomeXchange Consortium with the data set identifier PXD036791.

**File Name:** Supplementary Data 3

**Description:** O-glycosylated patches in the N/TERT-1 proteome. This table provides more detailed information on the identified glycan patches (>=5 O-glycans within 25 amino acids) within the N/TERT-1 proteome, listing specific sites, patch size (number of glycans), and patch length (distance from first glycan to last). Aditionally, a comparison is also made with previously described mucin domain-containing proteins from either the UniProt database (e.g., mucin-like or Pro/Thr/Ser-rich annotations) or from recent literature (Malaker et al. Nat. Comm. 2022)

**File Name:** Supplementary Data 4

**Description:** Quantitative proteomics comparing the N/TERT-1 GALNT KOs vs. WT cells. TMT-based quantitative proteomics was performed for each of the GALNT KOs on three technical replicates of the N/TERT-1 WT cells, as well as three different clones of the respective GALNT KO. Abundance ratios compared GALNT KO/WT. Sites on proteins with an abundance ratio <0.5 (blue) / >2 (red) were not included for further data analysis. MS data files are available via the ProteomeXchange Consortium with the data set identifier PXD031998.

**File Name:** Supplementary Data 5

**Description:** O-GalNAc glycan site occupancy in a native background. Glycan site occupancy was determined in the total cell lysate and the secretome of two N/TERT-1 samples: N/TERT-1 WT cells and N/TERT-1 cells with a knockout of the C1GALT1C1 gene (COSMC). Each sample was prepared in three technical replicates and fractionated into four fractions by high-pH C18 solid phase extraction. The MS/MS data of the COSMC KO cells was searched for the complete proteome, carrying 0 to 5 HexNAcs per peptide on Ser or Thr residues. The MS/MS data from the WT material was searched for the glycoproteins identified in the COSMC KO material, in combination with 17 N/TERT-1 O-GalNAc glycan compositions as described in De Haan et al. (Anal. Chem. 2022). When at least one glycoforms of a peptide was identified in the MS/MS search (Byonic score > 200 and manually confirmed), the presence of the non-glycosylated peptide, as well as the alternatively glycosylated peptides were evaluated in the MS1 data, based on accurate mass (+/- 1.5 ppm), retention time (within 5 min) and isotopic pattern (IDOTP > 0.85). The (glyco)peptides were relatively quantified based on the areas under the curve of the extracted ion chromatograms of the relevant charge states. MS data files are available via the ProteomeXchange Consortium with the data set identifier PXD031998.

**File Name:** Supplementary Data 6

**Description:** Literature comparison of O-GalNAc glycoprotein identifications. A comparison was made between the qualitative glycoprotein data of eight studies focusing on the precision mapping of the O-GalNAc glycoproteome, including the current study. The individual glycoproteins identified in the eight studies are listed, as well as their overlapping and unique identifications. The literature data was derived from Schjoldager et al. (EMBO R. 2015), Steentoft et al. (EMBO J. 2013), King et al. (Blood Ad. 2017), Bagdonaite et al. (EMBO R. 2020), Narimatsu et al. (Mol. Cell. Prot. 2019), Campos et al. Mol. Cell. Prot. 2015) and Yang et al. (Mol. Sys. Bio 2018).

**File Name:** Supplementary Data 7

**Description:** Description of the original data files uploaded to the ProteomeXchange Consortium via the PRIDE partner repository with the dataset identifier PXD031998.
