# Supplementary material for: Global mapping of GalNAc-T isoform-specificities and O-glycosylation site-occupancy in a tissue-forming human cell line
Source: Nat Commun. 2022 Oct 21;13:6257. doi: 10.1038/s41467-022-33806-8 (PMC9587226; doi:10.1038/s41467-022-33806-8)
Supplement: Supplementary file 11 — Reporting summary [file 41467_2022_33806_MOESM11_ESM.pdf]

nature portfolio

Corresponding author(s): Hans H. Wandall

Last updated by author(s): Sep 21, 2022

# Reporting Summary

Nature Portfolio wishes to improve the reproducibility of the work that we publish. This form provides structure for consistency and transparency in reporting. For further information on Nature Portfolio policies, see our Editorial Policies and the Editorial Policy Checklist.

## Statistics

For all statistical analyses, confirm that the following items are present in the figure legend, table legend, main text, or Methods section.

| n/a | Confirmed | |
|---|---|---|
| ☐ | ☒ | The exact sample size (*n*) for each experimental group/condition, given as a discrete number and unit of measurement |
| ☐ | ☒ | A statement on whether measurements were taken from distinct samples or whether the same sample was measured repeatedly |
| ☐ | ☒ | The statistical test(s) used AND whether they are one- or two-sided<br>*Only common tests should be described solely by name; describe more complex techniques in the Methods section.* |
| ☒ | ☐ | A description of all covariates tested |
| ☐ | ☒ | A description of any assumptions or corrections, such as tests of normality and adjustment for multiple comparisons |
| ☐ | ☒ | A full description of the statistical parameters including central tendency (e.g. means) or other basic estimates (e.g. regression coefficient) AND variation (e.g. standard deviation) or associated estimates of uncertainty (e.g. confidence intervals) |
| ☐ | ☒ | For null hypothesis testing, the test statistic (e.g. *F*, *t*, *r*) with confidence intervals, effect sizes, degrees of freedom and *P* value noted<br>*Give P values as exact values whenever suitable.* |
| ☒ | ☐ | For Bayesian analysis, information on the choice of priors and Markov chain Monte Carlo settings |
| ☒ | ☐ | For hierarchical and complex designs, identification of the appropriate level for tests and full reporting of outcomes |
| ☒ | ☐ | Estimates of effect sizes (e.g. Cohen's *d*, Pearson's *r*), indicating how they were calculated |

*Our web collection on statistics for biologists contains articles on many of the points above.*

## Software and code

Policy information about availability of computer code

| | |
|---|---|
| Data collection | Orbitrap Fusion Tune Application 3.1.2412.25 (mass spectrometry data collection on Oribitrap Fusion instrument)<br>Orbitrap Fusion Lumos Tune Application 3.3.2782.34 (mass spectrometry data collection on Oribitrap Fusion Lumos instrument) |
| Data analysis | Peak Scanner Software V1.0 - Thermo Scientific. (knockout clone screening using indel detection by amplicon analysis (IDAA))<br>Proteome Discoverer (2.3) using Sequest HT - Thermo Scientific. (mass spectrometry data processing)<br>Skyline (21.1.0.146) - ProteoWizard. (occupancy determination)<br>Prism (9.3.1) - GraphPad. (data visualization and linear regression calculation)<br>Excel 2016 - Microsoft. (glycan site-mapping on proteins and glycan patch identifications) |

For manuscripts utilizing custom algorithms or software that are central to the research but not yet described in published literature, software must be made available to editors and reviewers. We strongly encourage code deposition in a community repository (e.g. GitHub). See the Nature Portfolio guidelines for submitting code & software for further information.

## Data

Policy information about availability of data

All manuscripts must include a data availability statement. This statement should provide the following information, where applicable:

- Accession codes, unique identifiers, or web links for publicly available datasets
- A description of any restrictions on data availability
- For clinical datasets or third party data, please ensure that the statement adheres to our policy

All the MS data files are available via the ProteomeXchange Consortium67 with the data set identifier PXD036791 [http://proteomecentral.proteomexchange.org/cgi/GetDataset?ID=PXD036791] (see Supplementary Data 7 for raw MS data overview). The source data underlying the figures can be found in the source data file associated to the article.

## Human research participants

Policy information about studies involving human research participants and Sex and Gender in Research.

| | |
|---|---|
| Reporting on sex and gender | N/A |
| Population characteristics | N/A |
| Recruitment | N/A |
| Ethics oversight | N/A |

Note that full information on the approval of the study protocol must also be provided in the manuscript.

# Field-specific reporting

Please select the one below that is the best fit for your research. If you are not sure, read the appropriate sections before making your selection.

☒ Life sciences ☐ Behavioural & social sciences ☐ Ecological, evolutionary & environmental sciences

For a reference copy of the document with all sections, see nature.com/documents/nr-reporting-summary-flat.pdf

# Life sciences study design

All studies must disclose on these points even when the disclosure is negative.

| | |
|---|---|
| Sample size | In order to acquire the statistical power needed for significance testing, three biological (or technical in the case for wild type cells where biological replicates are not available) clone replicates were used. Samples for diffenretial glycoproteomics were muliplexed using TMTsixplex reagents, which allows 6 different ion reporter channels fitting three control (wildtype) samples and three test samples (GALNT knockout replicates). |
| Data exclusions | No samples were excluded from the data analysis.<br>For analysis of GalNAc-T isoform specific glcosylation, multi-glycan site peptides were excluded, because the quantification of these peptides may be unreliable due to missing information of all possible glycosylated species. |
| Replication | No independent replication of the complete experiments was performed. The reliability of the data was validated based on comparisons with literature, using comparable experimental approaches. Parts of our study replicated previously described findings, ensuring reliability of the new discoveries. Additionally, three biological replicate clones were used for each GALNT knockout, accounting for biological variation.<br>For occupancy determination, three technical replicates were used for secretome samples from both wild type and C1GALT1C1 knockout cells. In all cases, the estimated variation in occupancy between replicates was within 10%. |
| Randomization | N/A |
| Blinding | N/A |

# Reporting for specific materials, systems and methods

We require information from authors about some types of materials, experimental systems and methods used in many studies. Here, indicate whether each material, system or method listed is relevant to your study. If you are not sure if a list item applies to your research, read the appropriate section before selecting a response.

## Materials & experimental systems

| n/a | Involved in the study | |
|---|---|---|
| ☐ | ☒ | Antibodies |
| ☐ | ☒ | Eukaryotic cell lines |
| ☒ | ☐ | Palaeontology and archaeology |
| ☒ | ☐ | Animals and other organisms |
| ☒ | ☐ | Clinical data |
| ☒ | ☐ | Dual use research of concern |

## Methods

| n/a | Involved in the study | |
|---|---|---|
| ☒ | ☐ | ChIP-seq |
| ☒ | ☐ | Flow cytometry |
| ☒ | ☐ | MRI-based neuroimaging |

# Antibodies

| | |
|---|---|
| Antibodies used | mouse anti-Cytokeratin 10 / anti-K10 (IF 1:100). Dako (Agilent), Cat# M7002<br>rabbit anti-Involucrin (IF 1:200). Thermo Fisher, Cat# PA1-37934; RRID:AB_2265014<br>goat anti-mouse IgG (H+L) Antibody, AlexaFluorTM488 Conjugated (IF1:500). Molecular Probes, Cat# A-11029; RRID:AB_138404<br>goat anti-rat IgG (H+L) Antibody, AlexaFluorTM488 Conjugated (IF1:500). Molecular Probes, Cat# A-11006; RRID:AB_141373 |
| Validation | Anti-K10: This antibody was verified using Western blotting of cytoskeletal preparations from human epidermis. Here, the antibody labels a single 56.5 kDa band corresponding to CK 10. In two-dimensional immunoblotting of cytoskeletal proteins from human skin, the antibody labels a single dot corresponding to CK 10 (Validation statement from manufacturer's webstie).<br><br>Anti-Involucrin: This Antibody was verified by Relative expression to ensure that the antibody binds to the antigen stated (Validation statement from manufacturer's webstie). |

# Eukaryotic cell lines

Policy information about cell lines and Sex and Gender in Research

| | |
|---|---|
| Cell line source(s) | The N/TERT-1 cell line is derived from newborn male foreskin keratinocytes with transduced expression of hTERT and p16INK4a gene deletion (Dickson et al., Mol. Cell. Biol., 2000). This cell line was kindly provided by James G. Rheinwald's lab, Harvard Institute of Medicine, Brigham & Women's Hospital.<br>HEK293T (ATCC, Cat# CRL-3216) |
| Authentication | The cell lines have not undergone special authentication. All N/TERT-1 cell lines used were able to differentiate and grown organotypic 3D tissue cultures. |
| Mycoplasma contamination | The N/TERT-1 and HEK293T cells used are regularly tested for mycoplasma contamination. All tests have been negative. |
| Commonly misidentified lines<br>(See ICLAC register) | No commonly misidentified cell lines were used in this study |
